# A Cohort Study on Meniscal Lesions among Airport Baggage Handlers

**DOI:** 10.1371/journal.pone.0157336

**Published:** 2016-06-14

**Authors:** Sigurd Mikkelsen, Charlotte Brauer, Ellen Bøtker Pedersen, Tine Alkjær, Henrik Koblauch, Erik Bruun Simonsen, Karin Helweg-Larsen, Lau Caspar Thygesen

**Affiliations:** 1 Department of Occupational and Environmental Medicine, Bispebjerg University Hospital, Copenhagen, Denmark; 2 Department of Neuroscience and Pharmacology, University of Copenhagen, Panum Institute, Copenhagen, Denmark; 3 National Institute of Public Health, University of Southern Denmark, Copenhagen, Denmark; Le Fe Health Research Institute, SPAIN

## Abstract

Meniscal lesions are common and may contribute to the development of knee arthrosis. A few case-control and cross-sectional studies have identified knee-straining work as risk factors for meniscal lesions, but exposure-response relations and the role of specific exposures are uncertain, and previous results may be sensitive to reporting and selection bias. We examined the relation between meniscal lesions and cumulative exposure to heavy lifting in a prospective register-based study with complete follow-up and independent information on exposure and outcome. We established a cohort of unskilled men employed at Copenhagen Airport or in other companies in the metropolitan Copenhagen area from 1990 to 2012 (the Copenhagen Airport Cohort). The cohort at risk included 3,307 airport baggage handlers with heavy lifting and kneeling or squatting work tasks and 63,934 referents with a similar socioeconomic background and less knee-straining work. Baggage handlers lifted suitcases with an average weight of approximately 15 kg, in total approximately five tonnes during a 9-hour workday. The cohort was followed in the National Patient Register and Civil Registration System. The outcome was a first time hospital diagnosis or surgery of a meniscal lesion. Baggage handlers had a higher incidence of meniscal lesions than the referents. Within baggage handlers spline regression showed that the incidence rate ratio was 1.91 (95% confidence interval: 1.29–2.84) after five years as a baggage handler and then decreased slowly to reach unity after approximately 30 years, adjusted for effects of potential confounders. This relation between baggage handling and meniscal lesions was present for work on the apron which involves lifting in a kneeling or squatting position, but not in the baggage hall, which only involves lifting in standing positions. The results support that long-term heavy lifting in a kneeling or squatting position is a risk factor for the development of symptomatic meniscal lesions.

## Introduction

Musculoskeletal disorders and disability are among the leading causes of disease burden in high-income countries [1], and knee disorders including meniscal lesions constitute a large part of these disorders.

The prevalence of meniscal damage diagnosed by magnetic resonance imaging (MRI) was 30% among women and 42% among men in a general population of the United States of America, 50 to 90 years of age [2]. The incidence of meniscal tears is reported to be 2 per 1000 patients per year in Holland [3]. In England and Wales 25,000 yearly hospital admissions have been estimated to be due to meniscal tears [4]. Arthroscopic knee surgery for meniscal tears is one of the most common orthopedic surgical procedures in USA [5]. In 2011, the rate of meniscal surgery was 3.1 per 1000 persons in the Danish population [6].

In a recent systematic review and meta-analysis of risk factors for meniscal tears Snoeker et al. (2013) [3] identified age over 60, male gender, body mass index (BMI) >25 kg/m^2^, knee-straining sports, knee trauma, and occupational knee straining activities, in particular kneeling and squatting, as potential risk factors for meniscal tears. This assessment, however, was based on only a few studies. Some other factors have also been associated with meniscal lesions, e.g. systemic joint laxity [4]; knee joint laxity [7]; and signs of finger osteoarthritis and varus alignment of the knee [8].

A few epidemiological studies, three case-control studies [4,7,9] and two cross-sectional studies[10,11] have examined occupational risk factors, and have pointed to knee-straining work, in particular kneeling and squatting, as risk factors for meniscal lesions. Higher participation rates among cases compared to controls in the case-control studies and among exposed compared to non-exposed groups in the cross-sectional studies may have inflated the risk estimates. Furthermore, reporting bias with respect to self-reported work positions or meniscal lesions may also have resulted in biased risk estimates. Exposure-response relations were only sparsely examined in these studies.

The present study is a large register-based prospective follow-up study of a cohort of airport baggage handlers compared to a reference cohort. It examines the association of baggage handling and cumulative years as a baggage handler with first time hospital treatment for meniscal lesions in the period from 1990 to 2012. Follow-up is complete, and sources of information on exposure and outcome are independent.

## Material and Methods

In 2012 we established a historical cohort of unskilled male workers from electronic employee files of two baggage handling companies at Copenhagen Airport and from electronic member files of the local union of unskilled workers that organized baggage handlers in the airport. The files were established in 1983 (workers union) and in 1990 and 1995 (companies) and included former and present workers and historical information about entry and exit dates for work periods in specific departments, type of work, job titles, collective agreement codes and similar work task proxies by which baggage handling work and other work tasks could be identified for each calendar year since first entry date. Persons who had ever worked with baggage handling were classified as baggage handlers and those who never had as referents. The positive predictive value for concordance between company registration and union member registration as a baggage handler was 87%. In case of conflicting information we used the employer information which we considered as the most reliable and accurate because it was linked to payment of salary.

The reference group was supplemented with male members of two other local unions of unskilled workers covering the greater Copenhagen area, using the same electronic files and historical data as the local union that organized unskilled airport workers. In addition, we included male guard and security personnel from the Copenhagen Airport employee files and from the member files of the worker’s union that organized this staff. These files were established as electronic in 1990 and 1979, respectively, and contained similar information as the files described above. Thus, the reference cohort consisted of former and present unskilled workers with a variety of different tasks within the airport (e.g. guards and security personnel, area maintenance, cleaning, firefighting) and outside the airport but within the greater Copenhagen area (e.g. municipal workers, drivers, postal workers, garbage collectors, factory workers). From the cohort defined by company or union memberships as described above we excluded observations with invalid personal identification number, missing information on occupation, administrative/management/academic occupations, leave of absence, employment before 15 years of age, residence outside Denmark in the study period (1990–2012), employment after 31th December 2012, same entry and exit date, dead before employment and dead before 1990.

The cohort is named the Copenhagen Airport Cohort.

All Danish residents have a unique personal identification number which was used to identify persons in the files described above. We used this number to follow the cohort in the National Patient Register [12] for all contacts to the secondary health care system, the Civil Registration System [13] for information on mortality and migration, and registers at Statistics Denmark for information on pensioning and educational level [14–16].

Furthermore, self-reported data about work, health and lifestyle were collected by questionnaire in 2012. Questionnaires were delivered to all baggage handlers and a random sample of the reference cohort who met the following criteria: alive on April 2012, permanent residence in Denmark, aged 25–75 years, and had not previously declined to participate in research projects (an option in the Civil Registration System). Non-responders were contacted by phone. One questionnaire was sent to 3092 baggage handlers (2115 (68.4%) responded) and another to 2473 referents (1694 (68.5%) responded). Common for the two questionnaires were questions about height, weight, smoking, alcohol, and physical activity in leisure time (see supporting information S1 File. Questionnaires).

### Exposure

Basically, the work of airport baggage handlers consists of moving pieces of baggage from one place to another. The baggage handlers either work in the baggage sorting area inside the terminal building or outdoors on the apron.

In the baggage sorting area they load and unload baggage carts and baggage containers to or from a belt conveyer. The work is evenly distributed over the day. The work is performed standing. Pneumatic lifting hooks were introduced in 1998 and were used most of the time by 34%, sometimes by 37%, and seldom by 24% of the baggage handlers, according to our 2012-survey.

Work on the apron consists of work on the ground and work inside the airplane baggage compartments. On the ground the work is loading and unloading baggage carts to or from a belt conveyer to the baggage compartment opening. The work is standing. The belt conveyer is height adjustable. Inside the baggage compartment the work consists of lifting the baggage to or from the conveyer and to pack or unpack the baggage inside the compartment. Extended and flexible belt loaders at the aircrafts were gradually introduced from 2002 to 2005 and used routinely from 2004. They allow the baggage to be conveyed to any place in the compartment. Depending on the size of the compartment and conveyer belt system, loading and unloading work inside the compartment is done by one or two baggage handlers. Work positions depend on the height of the compartment, the height of the baggage handler and personal preferences, and may be standing, stooped, sitting, squatting and kneeling. Baggage handlers in the team changed between working on the ground and in the aircraft baggage compartment. Work on the apron is unevenly distributed over the day. A team of 2–4 baggage handlers normally loads or unloads one aircraft per hour (a handling). This takes 15–30 minutes of intensive work and is followed by other duties or a pause until the next handling.

Based on detailed employer and airport statistics the average weight of a baggage piece is approximately 15 kg, and the average of total baggage lifted per day is approximately five tonnes per baggage handler, slightly less in the baggage sorting area than on the apron. This daily lifting load has been rather constant over years since 1990.

In the analyses, we included two measures of exposure: work as a baggage handler (ever: yes/no) and cumulative years of employment as a baggage handler. The percentage of employment as a baggage handler was computed for each year and cumulated during follow-up resulting in the time-dependent cumulative years as a baggage handler available for each year. This cumulative exposure measure was also calculated separately for work in the baggage sorting area and on the apron before and after the introduction of lifting equipment and extended belt loaders in 1998 and 2004, respectively.

### Outcome

The outcome was first hospitalization with a meniscal disease as the primary discharge diagnosis or surgery for a meniscal lesion. Before 1994 diagnoses were recorded according to the International Classification of Diseases version 8 (ICD-8) and from 1994 according to version 10 (ICD-10). Before 1996 surgical procedures were coded according to a Danish national classification system, and from 1996 by the Nomesco Classification of Surgical Procedures.

We included the following diagnoses and surgical procedures as meniscal lesions: ICD-8: Old meniscal disease (724.19), knee distorsion with meniscal lesions (844.02, 844.03, 844.04), traumatic lesion of menisci (849.45, 849.46, 849.48, 849.49); ICD-10: old traumatic meniscal lesion (M23.2), other meniscal disorders (M23.3), traumatic meniscal rupture (S83.2) and traumatic lesion of menisci and ligaments (S83.7C); Danish surgical procedure classification codes before 1996: resection of meniscus (72540, 72541, 72549, 74560, 74570), meniscectomy (72640, 72641, 72649, 74580, 74590,74600, 74610), reinsertion of meniscus (72740, 72741, 72749, 74620, 74630); Nomesco-codes from 1996: operations on menisci of the knee (NGD-group).

### Potential Confounders

We considered the following factors as potential confounders of the relation between meniscal lesions and working as a baggage handler and cumulative years as a baggage handler: age, calendar year, highest attained educational level, pre-employment knee-trauma and -surgery, availability of lifting equipment in the baggage sorting area and extended belt loaders in aircraft baggage compartments.

Age was included categorically (<30, 30–44, 45–59 and 60+ years) and as a continuous linear effect; calendar year by categories 1990–1994, 1995–1999, 2000–2004 and 2005–2012; and education by categories elementary school, high school, vocational education, and higher education. Information about pre-employment knee-trauma and -surgery was obtained from the National Patient Register based on discharge diagnoses of knee luxations, distorsions and traumatic lesions of ligaments and menisci (ICD-8: 836, 844, 849.40–849.49; ICD-10: S83) and surgical procedures on the knee joint, ligaments and capsule (Danish surgical procedure classification codes before 1996: 71540 to 74549 for the knee region; Nomesco codes from 1996: groups NGA to NGK).

Use of lifting equipment in the baggage sorting area was included as a binary variable defined by the year when this aid was introduced (0 = before 1998, 1 = 1998 and after) and similarly for extended belt loaders in the airplane baggage compartments (0 = before 2004, 1 = 2004 and after).

From the 2012 survey we report BMI (weight(kg)/height(m)^2^), smoking, alcohol consumption, and physical activity in leisure time as additional information about the comparability of baggage handlers and referents.

### Statistical analysis

From the basic cohort of 69,175 persons we excluded persons with an outcome before first date of employment leaving 67,241 persons in the cohort. They were followed from start of first employment, January 1990 or immigration after employment, whichever came last, and until first diagnosis or surgery for a meniscal lesion, emigration, death or end of follow-up (31 December 2012), whichever came first.

The effect of baggage handling was examined in four preselected models with the following baggage handling covariates.

1. Baggage handlers compared to the reference group.
2. Baggage handler cumulative years categorical (reference group, 0.1–2.9 years, 3.0–9.9 years, 10.0–19.9 years, and 20+ years).
3. Cumulative years as a continuous variable and the binary group variable, coded ‘1’ for referents and ‘0’ for baggage handlers. By this coding, the effect of cumulative years only refers to baggage handler cumulative years.
4. Cumulative years as a restricted cubic spline with knots at 5th, 27.5th, 50th, 72.5th, and 95th percentiles of the distribution of cumulative years. We used the PSPLINET macro. In this model we included the same binary group variable, so the effect of cumulative years only refers to baggage handler cumulative years. Model 4 included test for non-linearity of the influence of cumulative years on meniscal lesions using the likelihood ratio test, comparing the model with only the linear term to the model with the cubic spline terms. In the case that a linear effect was not accepted we examined a piecewise linear spline based on a visual analysis of the form of the restricted cubic spline (see the result section).

These models offer insight from different angles into the association between baggage handling and meniscal lesions and thereby yields a more comprehensive basis for interpretation of the results. It may also be noted that in the analyses of dose-response relations the reference group only serves as an anchor point for comparison with baggage handlers with short exposure. This contrast is assessed with the coding of the reference group as ‘1’ and the baggage handlers as ‘0’, and this coding also secures that the reference group is not included as a zero-exposed group in the assessment of a dose-response among baggage handlers. The analyses were performed unadjusted and adjusted for effects of potential confounders.

We also estimated the influence of cumulative years in the baggage sorting area and on the apron, and before and after introduction of a lifting aid and extended belt loaders, respectively. We included cumulative years as categorical variables with the same categories as above, except for the latter analyses where we used categories 0.1–2.9, 3.0–5.9 and 6.0+ because of few cases for higher categories of cumulative years.

Finally, we evaluated the influence of years since termination of employment in a model adjusted for confounders and cumulative years. The influence of years since termination was modelled as a restricted cubic spline. We also tested whether there was an interaction between cumulative years and years since termination, included as linear effects.

As sensitivity analyses we repeated the analyses for models 1 to 4 on outcomes that occurred after the change of diagnostic classification system from ICD-8 to ICD-10 in 1994, and for outcomes coded as a traumatic lesion or rupture (ICD-8: 844 and 849, ICD-10: S83.2 and S83.7C), and for other diseases (ICD-8: 724.19, ICD-10: M23.2 and M23.3).

Two-sided p values below 0.05 were considered statistically significant. Data were analyzed using Poisson regression with log-transformation of person-years at risk and Cox regression (restricted cubic spline) using SAS version 9.3 (SAS Institute Inc, Cary, North Carolina, USA).

### Ethics Statement

The study was notified to the Scientific Ethical Committee, The Capital Region of Denmark (journal no.: H-4-2011-125), but according to Danish law, register-based studies and questionnaire studies need neither approval from ethical and scientific committees nor informed consent [17], and was not obtained. The study was registered at The Danish Data Protection Agency [18]. All records/information was anonymized and de-identified prior to analysis.

## Results

The cohort consisted of 3,307 baggage handlers and 63,934 referents at risk of a first time meniscal lesion in the observation period from 1990 to 2012 (Table 1). At entry to the cohort baggage handlers were younger, and had vocational education more often than the referents. Self-report data from 2012 showed similar BMI, smoking habits, weekly alcohol consumption and leisure time physical activity.

**Table 1:** Baseline descriptives for persons at risk of a first time meniscal lesion. Copenhagen Airport Cohort, by baggage handlers and referents.

		Baggage handlers		Reference group	
***Register based factors***^***1***^		n	%	n	%
Total participants		3,307	100	63,934	100
Age	<30	2,044	62	29,605	46
	30–44	1,151	35	20,597	32
	45–59	110	3	9,888	15
	60+	2	0	3,844	6
Educational level	Elementary school	1,511	46	36,396	57
	High school	426	13	8,314	13
	Vocational education	1,229	37	16,013	25
	Higher education	141	4	3,211	5
Pre-employment knee trauma		172	5	2,149	3
***Self-reported factors*, *survey 2012***					
Total participants		1,703	100	1,909	100
Body mass index	<18.5	1	0	9	0
	18.5–24.9	576	34	672	36
	25–29.9	826	49	871	46
	30.0+	280	17	325	17
Smoking	Never	650	38	654	34
	Past	581	34	656	35
	Current	459	27	588	31
Alcohol (units of 12 grams per week)	None	413	25	494	26
	1–21	1,191	71	1,272	67
	>21	78	5	125	7
Physical activity in leisure time	Sendentary	164	10	248	13
	Low	599	36	677	36
	Medium	685	41	691	37
	High	234	14	269	14

^1^ Descriptive statistics for the first year during follow-up that a person was a baggage handler, or first year during follow-up for workers who were never a baggage handler (reference group).

There were 3,520 incident cases (3,243 referents and 277 baggage handlers) of which 3,131 cases were identified by a meniscal discharge diagnosis and 885 by meniscal surgery, with an overlap of 496 cases. Among cases defined by diagnosis, 3,062 (98%) had a diagnosis indicating a traumatic lesion or rupture of a meniscus. The vast majority of these cases were ICD-10 cases M23.2 (old traumatic meniscal lesion) and S83.2 (traumatic meniscal rupture) with 1,511 and 1,150 cases, respectively. No cases were classified as meniscal degeneration (M23.3B). Among the 885 surgical cases, 656 (74%) had arthroscopic meniscectomy or meniscal resection or reinsertion.

At the group level, the referents had a significantly lower risk of a meniscal lesion than the total group of baggage handlers (Model 1, Table 2), but the difference became smaller and non-significant when compared to baggage handlers with 0.1–2.9 cumulative years as baggage handlers (Model 2, Table 2) and the difference almost disappeared in the linear spline model (Model 4, Table 2). In this model, the baggage handler group refers to baggage handlers when they start as baggage handlers (cumulative years = 0).

**Table 2:** Association between status as a baggage handler and cumulative years as a baggage handler and meniscal lesions. Copenhagen Airport Cohort, 1994–2012.

Model	Cases	Person-years	IR	IRR, unadjusted (95%CI)	IRR,adjusted^1^ (95% CI)
***1*. *Baggage handler***					
No	3,243	949,530	341.5	0.55 (0.49–0.62)	0.64 (0.52–0.80)
Yes	277	44,583	621.3	1.00 (ref)	1.00 (ref)
p-value				< .0001	< .0001
***2*. *Baggage handler years*, *categorical***					
Non baggage handler	3,243	949,530	341.5	0.61 (0.49–0.76)	0.79 (0.60–1.04)
0.1–2.9 years	86	15,371	559.5	1.00 (ref)	1.00 (ref)
3.0–9.9 years	107	15,242	702.0	1.25 (0.94–1.67)	1.38 (1.04–1.83)
10.0–19.9 years	66	9,882	667.9	1.19 (0.87–1.65)	1.40 (1.01–1.94)
20.0+ years	18	4,087	440.4	0.79 (0.47–1.31)	1.02 (0.61–1.71)
p-value (df = 4)				< .0001	0.0003
p-value (only baggage handlers, df = 3)				0.15	0.16
***3*. *Baggage handler years*, *linear***^***2***^					
Baggage handler					
No				0.51 (0.43–0.61)	0.66 (0.52–0.83)
Yes				1.00 (ref)	1.00 (ref)
Continuous linear (per 5 years)				0.96 (0.88–1.04)	1.01 (0.93–1.10)
p-value				0.29	0.76
***4*. *Baggage handler years*, *linear spline***^***2***^					
Baggage handler					
No				0.71 (0.53–0.94)	0.95 (0.68–1.34)
Yes				1.00 (ref)	1.00 (ref)
Continuous linear 0–4.9 years (per 5 years)				1.70 (1.15–2.52)	1.91 (1.29–2.84)
p-value				0.0079	0.0012
Continuous linear 5+ years (per 5 years)				0.84 (0.75–0.95)	0.88 (0.78–1.00)
p-value				0.0063	0.043
p-value of no difference between linear slopes				0.035	0.020

Abbreviations: IR, incidence rate per 100,000 person-years; IRR, incidence rate ratio.

^1^ Adjusted for age, calendar year, use of baggage lifter, use of baggage belt loader, educational level and pre-employment knee injuries.

^2^ Also adjusted for baggage handler (yes/no).

The incidence rate (IR) for baggage handlers with 3.0 to 9.9 and 10.0–19.9 cumulative years was approximately 1.40 times higher than for the baggage handlers with 0.1–2.9 years, while the IR for the category 20+ years was approximately the same (Model 2, Table 2). The difference between the reference group and the four baggage handler groups was highly significant, but the difference between the four baggage handler groups was not (p = 0.16). With the inverse U-shaped pattern of incidence rate ratios (IRR) by categories of cumulative years it is not surprising that a continuous linear effect of cumulative years was not significant (model 3 in Table 2).

**Fig 1:**
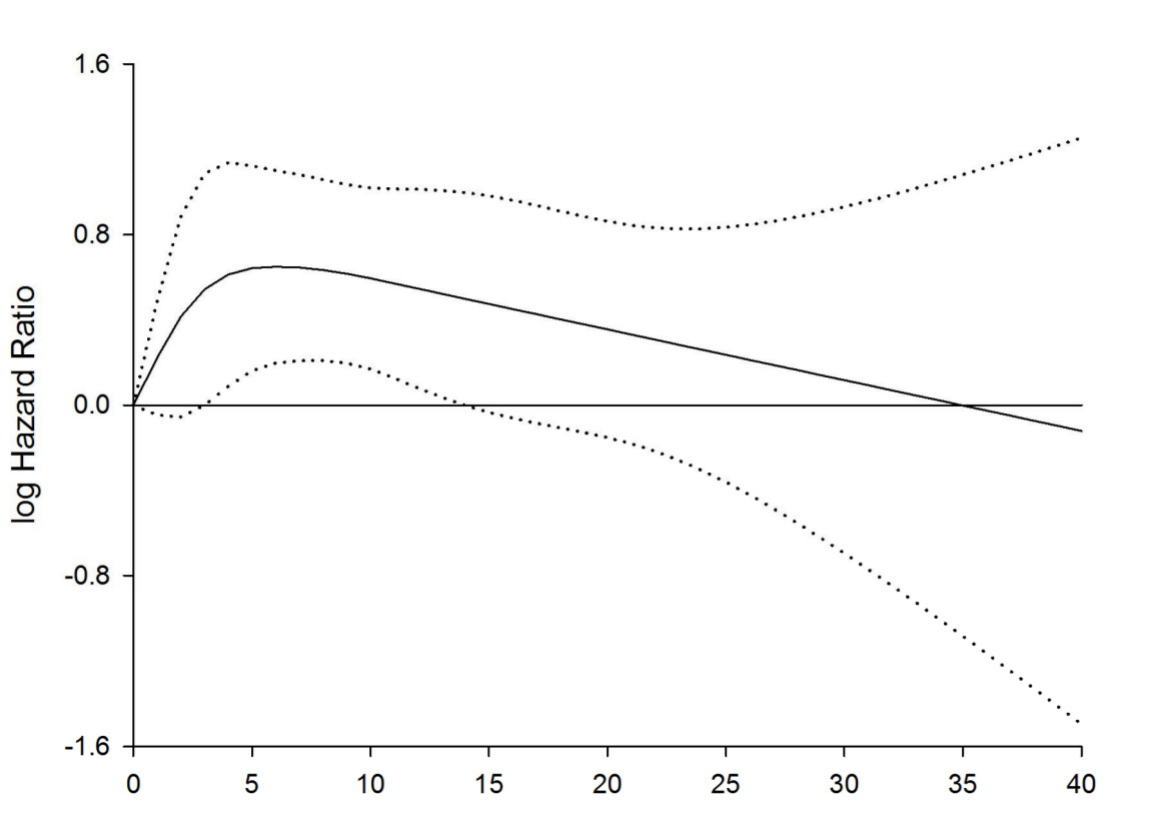
Restricted cubic spline graph of the association between cumulative years as a baggage handler and meniscal lesions. **Copenhagen Airport Cohort, 1994–2012.** Cox regression model adjusted for status as a baggage handler (yes/no), age, educational level, calendar year, pre-employment knee injury, use of baggage lifter and use of extendable belt loader. Dotted lines represent 95% confidence intervals.

The restricted cubic spline analyses with cumulative years as a continuous variable confirmed that the risk of meniscal lesions among baggage handlers increased during the first years of employment and then decreased, and these effects were significantly different from a simple linear effect (p = 0.023). The inflexion point seemed to be close to 5 years (Fig 1). A linear spline with 5 years as the inflexion point revealed a statistically significant increase during the first 5 years of employment (IRR = 1.91 (95%CI: 1.29–2.84), p = 0.0012) and then a slower decline (IRR = 0.88 for a five year increase in cumulative years (95%CI: 0.78–1.00), p = 0.043). The difference between the two linear slopes was significant (p = 0.020) (Model 4, Table 2). The latter slope was modelled with reference to unity and implies that a decline to unity from an IRR = 1.91 after the first five years will take another 25 years.

In the analyses stratified by work in the baggage sorting area and on the apron, cumulative years in the baggage sorting area had no association with meniscal lesions, neither before nor after introduction of lifting equipment in 1998 (data not shown). For work on the apron, the effect of cumulative years was very similar to the results for the whole material shown in Table 2 (model 2) (3.0–9.9 years: IRR = 1.58 (95%CI: 1.17–2.14); 10.0–19.9 years: IRR = 1.37 (95%CI: 0.98–1.92); 20.0+ years: IRR = 1.03 (95%CI: 0.72–1.46)). Before the introduction of extendable beltloaders in 2004 the IRR for the 3–5.9 cumulative years on the apron was 1.56 (95%CI: 1.14–2.13) and after 2004 it was 1.21 (95%CI: 0.74–1.98). For 6.0+ cumulative years the IRR was close to unity for both time periods. Thus, the pattern of increasing and then decreasing risk of meniscal lesions associated with cumulative years found in the main analyses was due to cumulative years of work on the apron, mainly before 2004.

There was no effect of time since termination of employment on the risk of meniscal lesions and no interaction between time since termination and cumulative years (data not shown).

Sensitivity analyses (only outcomes from 1994, traumatic lesion diagnoses and other diagnoses) showed a similar pattern of effects of cumulative years as described for the main analysis (data not shown).

The differences between the unadjusted and adjusted IRR-estimates were small. Although the correlation between age and cumulative years as a baggage handler was high (Pearsons’ correlation coefficient = 0.63) effects of cumulative years with and without adjustment for age were quite similar (data not shown).

The following adjusted effects were found for potential confounders (model 2, Table 2): the risk of meniscal lesions decreased with increasing age, especially in the oldest age group (30–44 years: IRR = 1.02 (95% CI: 0.93–1.12); 45–59 years: IRR = 0.91 (95% CI: 0.82–1.01); 60 years or older: IRR = 0.28 ((95% CI: 0.23–0.34), compared to being younger than 30 years, p<0.0001); increased with increasing calendar year (IRR per 10 years = 1.33 ((95% CI: 1.26–1.41), p<0.0001); increased with pre-employment knee-injury (IRR = 2.75 (95% CI: 2.41–3.12), p<0.0001); and differed significantly between educational groups (high school: IRR = 0.90 (95% CI: 0.81–1.00); vocational education: IRR = 1.32 (95% CI: 1.23–1.43); higher education: IRR = 0.89 (95% CI: 0.75–1.06), compared to elementary school, p<0.0001). Use of baggage lifter and belt-loader had no significant effects.

## Discussion

In this large cohort study the risk of meniscal lesions was significantly increased for baggage handlers compared to a reference group of other unskilled workers with a variety of different work tasks.

Within baggage handlers the risk of meniscal lesions increased with cumulative years during the first five years as a baggage handler and then slowly decreased to reach unity after approximately 30 years as a baggage handler. The risk associated with baggage handling was only found for work on the apron, and more pronounced the period before extendable belt loaders were introduced. The risk of meniscal lesions among baggage handlers after cessation of employment did not depend on the time since cessation or on cumulative years as a baggage handler before cessation.

### Exposure

We chose a reference group of unskilled workers to secure socioeconomic and sociocultural comparability between the two groups. However, this choice was at the cost of a maximal contrast in knee-straining work, because many unskilled workers have some knee straining work. On average, however, we believe that there was a significant contrast in the lifting and knee-straining work positions between the two groups, and even if the choice of referents may have sacrificed some contrast, this does not influence effects of cumulative years as a baggage handler. Also, there may be some misclassification of exposure because the status as a baggage handler was derived from job title and employment in certain departments with mainly baggage handling tasks and may have included some persons with other tasks (e.g. tractoring aircraft, deicing, special services etc.). Thus, among the participants defined as baggage handlers in the registers, 9.6% of those who had participated in the baseline survey declared that they had not worked as baggage handlers in the airport. Finally, even if baggage handling only involves relatively few homogeneous work tasks, cumulative years is still a crude measure of cumulative exposure and could therefore bias our results towards null.

Our results are consistent with other studies using occupation or job title as the exposure contrast [4,7,9,10,19,20]}. The role of cumulative exposure of knee straining work for meniscal lesions has only been examined in one other study [21]. In this study the prevalence of medial meniscal lesions, diagnosed by MRI, decreased with increasing seniority among floor layers with high seniority, consistent with our results, but the statistical significance of this finding was not reported.

### Outcome

Our outcome was based on hospital treatment for meniscal lesions registered in the Danish National Patient Register. The register is considered to be quite complete and of high quality ([12]. A quality assessment study found that 83% of orthopedic diagnoses and 92% of orthopedic surgical procedures reported to the register were correct [22].

Almost all diagnoses of meniscal lesions were considered to have a traumatic origin, either as an ‘old traumatic lesion’ or as a ‘traumatic rupture’. The age distribution of the two diagnostic groups was comparable (data not shown). No meniscal disease was diagnosed as meniscal degeneration, and surgical procedure codes did not reflect meniscal pathology. Thus, we could not analyze diagnostic subgroups as acute traumatic or degenerative lesions [4,23,24]. Sensitivity analyses of diagnoses since 1994, and of traumatic rupture diagnoses and other diagnoses separately, showed similar patterns of association with baggage handling as in the main analyses.

We could not differentiate between medial and lateral meniscal lesions. Some studies indicate that the lateral meniscus is less sensitive to knee-straining work [7,19]. Our results may therefore underestimate the effect of baggage handling on medial meniscal lesions. Also, we do not know if the meniscal lesion was on the right of left side, but we have no reason to suspect that the degree of knee straining work is different for the two sides.

We cannot exclude that the incidence of meniscal lesions is the same among baggage handlers and referents but become symptomatic more frequently among baggage handlers owing to their knee-straining work. One cross-sectional study, however, found meniscal lesions diagnosed by MRI more frequently among floor layers than among controls, regardless of knee symptoms [19]. Furthermore, people who are handicapped at work by knee symptoms may be more likely to seek medical advice and more likely to be referred to hospital treatment [9], which could spuriously inflate risk estimates. However, it seems unlikely that referral bias is associated with cumulative years.

### Confounding

Associations between measures of exposure and meniscal lesions were adjusted for age, educational level, calendar year and pre-employment knee-trauma or -surgery, including sport related lesions. Self-reported information on life style factors BMI, smoking, and leisure time physical activity did not indicate any major differences between baggage handlers and referents. We were not able to control for any differences in participation in knee-straining sports or knee abnormalities, e.g. knee joint laxity or varus alignment, or generalized osteoarthrosis or joint laxity which have been associated with an increased incidence of meniscal lesions [4,7,8]. However, considering the comparability of baggage handlers and referents in other aspects we find it unlikely that our results are explained by residual confounding related to these and other factors. A basic comparability of the two groups is further indicated by the finding that baggage handlers and referents had similar risks of meniscal lesions after adjustment for effects of cumulative years, modelled as a two piece linear spline. This finding implies that baggage handlers had the same risk of meniscal lesions as the referents when they started their work and speaks against a primary healthy-worker effect. Furthermore, if pre-employment knee or meniscal cartilage conditions were symptomatic one would expect that persons with such a condition would select jobs with less knee straining work than baggage handling. A secondary healthy-worker effect could have biased our results towards the null if more baggage handlers than referents with meniscal knee symptoms left their job and therefore avoided subsequent hospital treatment.

### Interpretation

The results for the linear spline model also touches on mechanisms of the occurrence of meniscal lesions. Since the incidence increased during the first years one may imagine that meniscal vulnerability to tears builds up with increasing cumulative years, for example by micro-traumas causing micro-lesions at a rate that exceeds the healing process. If one part of the population was sensitive to this mechanism while another was resistant the incidence of meniscal tears would increase during the first years of baggage handling as vulnerability to tears builds up in the sensitive part of the population, and then decrease because the proportion of sensitive persons at risk relative to resistant persons will become smaller as time passes and tears occur in the sensitive part. Thus, the observed pattern could possibly be explained by a higher vulnerability of subgroups of the population and a survivor effect. Vulnerability factors could be related to genetic, anatomic or functional characteristics of the knee joint and the menisci. In addition, the temporal development in the risk of meniscal lesions could be influenced by more overt accidents among newly hired workers.

It was suggested in an editorial on MRI findings among middle-aged and older floor layers that kneeling and squatting might accelerate degenerative effects of aging on the menisci [25]. This mechanism is not supported by our results. Most baggage handlers had cumulated 5 years as a baggage handler before the age of 45, and the IRR of meniscal lesions decreased slowly with more cumulated years. Furthermore, in our study the incidence rate of being hospitalized with a meniscal lesion decreased with increasing age. This may seem at odds with an increasing prevalence of meniscal lesions, diagnosed by MRI, found in a general population, 50 to 90 years old [2]. However, for chronic disorders the prevalence may increase with age even if the incidence decreases. Actually, in the same population, age was not a risk factor for the incidence of meniscal tears, diagnosed by MRI, during 30 months of follow-up [8].

If the association between baggage handling and meniscal lesions is causal it is most likely due to knee-straining work during lifting of baggage in a standing position or in a kneeling or squatting position. Since there were no associations between cumulative years of work in the baggage sorting area and meniscal lesions our data do not support that lifting of baggage in a standing position causes meniscal lesions. Meniscal lesions were statistically significantly associated with self-reported lifting or carrying of heavy weights in one but not significantly so in another case-control study using the same exposure measures [4,9]. The pattern of association between cumulative years of work on the apron and meniscal lesions was similar to the overall pattern and more pronounced before 2004. However, in the main analysis the effect of working on the apron before and after 2004 was not significantly different. Unfortunately, we did not have data at the individual level to split work on the apron into work on the ground and work in the baggage compartment. In the survey data almost all baggage handlers working on the apron indicated that they worked equally on the ground and in the baggage compartment, and in the baggage compartment they worked on average approximately 50% of the time in a kneeling or squatting position. We do not know, if kneeling or squatting work positions were more frequent before than after the introduction of extended conveyer belts introduced around 2004.

### Strengths and limitations

The major strengths of this study are the prospective cohort study design with complete follow-up; the large material; exposure information recorded before the outcome (constituting the study as a prospective follow-up study); and independent sources of information about exposure and outcome. The major limitations are potential misclassification of exposure and outcome, which may have biased the results towards no association; and the outcome includes only meniscal lesions severe enough to require hospital treatment.

## Conclusion

The incidence of meniscal lesions was higher among baggage handlers than among referents. This difference was explained by an increasing incidence with cumulative years as a baggage handler during the first approximately five years and then a slower decrease. The increased risk was found for work tasks with lifting in combination with kneeling and squatting, but not with lifting in only a standing position.

## Supporting Information

S1 File Questionnaires. (DOCX)

## Abbreviations

BMI: body mass index (weight(kg)/height(m)^2^)
ICD: international classification of diseases
IR: incidence rate
IRR: incidence rate ratio
