# Supplementary material for: A Cohort Study on Meniscal Lesions among Airport Baggage Handlers
Source: PLoS One. 2016 Jun 14;11(6):e0157336. doi: 10.1371/journal.pone.0157336 (PMC4907513; doi:10.1371/journal.pone.0157336)
Supplement: S1 File — (DOCX) [file pone.0157336.s001.docx]

**S1 File. Questionnaires**

**1. Questionnaire for baggage handlers**

1. Have you ever worked as a baggage handler
2. What year did you start as a baggage handler
3. Do you still work as a baggage handler
4. How many years have you worked as a baggage handler
5. Which handling companies have you worked in
6. Think about your work as a baggage handler, how often did you work the following hours

(response options day work, evening work, night work)

1. How often have you worked the following hours a day
2. How many years have you worked the following places as a baggage handler (response options: on the ramp domestic, on the ramp international, in the baggage hall domestic, in the baggage hall international
3. State in the scheme below what periods you mainly worked at the places mentioned (same response options as 8, detailed by calendar years
4. Have you ever worked as a baggage handler in the baggage hall
5. Have you been a foreman or have you had paper work in the baggage hall (start and stop year)
6. In which of the following halls did you work (different named halls)
7. How much of your time in the baggage hall did you work with the following: unloading, loding, odd size
8. Have you had the opportunity to use a lifting hook in the baggage hall
9. How often did you use a lifting hook when loading or unloading the following types of baggage to belly carts
10. Same as 15. …. to containers
11. When you used a lifting hook, why did you use it (various reasons)
12. When you did not use a lifting hook, why did you not use it (various reasons)
13. Have you ever worked as a baggage handler on the ramp
14. Have you been loadmaster or have you had paper work working on the ramp (start and stop year)
15. When you have loaded or unloaded slim body planes (planes without containers), did you work mostly inside the baggage compartment or outside the baggage compartment
16. How often have you worked in the following positions when you worked inside the baggage compartment (standing, stooped, squatting, kneeling, sitting, other)
17. Have you worked on the ramp with other tasks than baggage handling (de-icing, catering, cleaning, other, - number of years)
18. Did you , aside from working as a baggage handler, work in a cargo company
19. Have you had other work concurrent with your work as a baggage handler (hours per week)
20. How would you characterize your concurrent work (less, equal or harder physically than working as a baggage handler
21. How large a part of your whole working life have you indoors and outdoors, respectively
22. How is your health, all together (excellent, very good, good, fair, bad)
23. Do you think that you have contracted one or more diseases that may be caused by your work at Copenhagen Airport
24. How much have you been bothered by pain an discomfort in the following body parts during the previous 12 months
25. Have you ever had surgery in any of the following body parts (year of first operation)
26. When were you born
27. How high are you
28. What is your weight
29. What was your weight when you were 20 years old
30. Are you left-handed, right-handed, ambidexter
31. Do you smoke (no, previously, yes)
32. How many years did you smoke
33. How much do/did you smoke, on average per day
34. How much have you been exposed to passive smoking
35. How many units of alcohol do you usually consume per week
36. If you should assess your physical activity in leisure time including transport to and from work within the last year, which of the following groups would fit for you (descriptions of activity)
37. Have you practiced the following forms of physical activity within the last 12 months (different physical activities)
38. Do you want to add anything:

**2. Questionnaire for the control group**

1. How is your health, all together (excellent, very good, good, fair, bad)
2. How much have you been bothered by pain an discomfort in the following body parts during the previous 12 months
3. When were you born
4. How high are you
5. What is your weight
6. What was your weight when you were 20 years old
7. Do you smoke (no, previously, yes)
8. How many years did you smoke
9. How much do/did you smoke, on average per day
10. How much have you been exposed to passive smoking
11. How many units of alcohol do you usually consume per week
12. If you should assess your physical activity in leisure time including transport to and from work within the last year, which of the following groups would fit for you (descriptions of activity)
13. How large a part of your whole working life have you indoors and outdoors, respectively
14. Did you ever work at Copenhagen Airport (guard security, tractoring, catering, cleaning, service)
15. How large a proportion of the time you worked at Copenhagen Airport did you work indoors and outdoors?
16. If you have comments, write here:
